# Patterns of participation restriction among older adults at risk of falls and relationship with intrinsic capacity: A latent cluster analysis

**DOI:** 10.3389/fmed.2022.1023879

**Published:** 2022-11-25

**Authors:** Reshma Aziz Merchant, Yiong Huak Chan, Ivan Aprahamian, John E. Morley

**Affiliations:** ^1^Division of Geriatric Medicine, Department of Medicine, National University Hospital, Singapore, Singapore; ^2^Department of Medicine, Yong Loo Lin School of Medicine, National University of Singapore, Singapore, Singapore; ^3^Biostatistics Unit, Yong Loo Lin School of Medicine, National University of Singapore, Singapore, Singapore; ^4^Geriatrics Division, Department of Internal Medicine, Jundiai Medical School, Jundiai, São Paulo, Brazil; ^5^Division of Geriatric Medicine, Department of Medicine, Saint Louis University School of Medicine, St. Louis, MO, United States

**Keywords:** life-space mobility, participation restriction, latent class analysis, falls, intrinsic capacity

## Abstract

**Introduction:**

The concept of participation restriction was first described by the World Health Organization in 2001 as a component of The International Classification of Functioning, Disability and Health Framework. Both falls and fear of falling (FOF) are associated with social isolation, depression, anxiety, poor quality of life and cognitive impairment resulting in participation restriction. Life-space mobility (LSM) is an important indicator for participation restriction which depends on multiple inter-related factors. We aimed to determine participation patterns using latent cluster analysis (LCA) in older adults at risk of falls, its relationship with intrinsic capacity (IC) and its risk prediction.

**Methods:**

Cross-sectional study of 154 community dwelling older adults ≥ 60 years with falls or risk of falls was conducted. Questionnaires were administered on demographics, hearing, LSM, frailty (FRAIL scale), anorexia of aging (SNAQ), cognition (Montreal Cognitive Assessment, MoCA), FOF (Falls Efficacy Scale-International), physical function, and assessment for handgrip strength (HGS), gait speed, 5-times sit to stand (STS), vision and times-up-and-go (TUG) were performed. Six IC domains (vision and hearing, cognition, nutrition, mobility and depression) were measured.

**Results:**

Three pattern of participation cluster were identified, high (*n* = 63, 40.9%), moderate (*n* = 83, 53.9%) and low (*n* = 8, 33 5.2%). Individuals in the high participation cluster were significantly younger, had higher LSM scores and lower FES-I scores, more robust, fewer ADL and IADL limitations, lower prevalence of low HGS, higher gait speed and shorter TUG. In the fully adjusted model compared to the high participation cluster, moderate participation was significantly associated with low MoCA scores (OR 4.2, 95% CI 1.7–10.4, *p* = 0.02), poor STS (OR 7.1, 95% CI 3.0–17.0, *p* < 0.001) whereas low participation was associated with anorexia of aging (OR 9.9, 95% CI 1.6–60.9, *p* = 0.014), poor STS (OR 19.1, 95% CI 2.0–187.5, *p* = 0.011) and hearing impairment (OR 9.8, 95% CI 1.4–70.8, *p* = 0.024). Participants with 3 out of 6 IC decline had a probability of greater than 80% to belong to the low/moderate participation class.

**Discussion:**

Physical function, cognition, hearing and nutrition were significantly associated with low and/or moderate participation class. Future studies are needed to evaluate improvement in participation of those with falls or at risk for falls through restoration of IC.

## Introduction

The concept of participation restriction was first described by the World Health Organization (WHO) in 2001 as a component of The International Classification of Functioning, Disability and Health (ICF) Framework (1, 2). The ICF is a classification system which describes health status, disability, and functioning in the context of surrounding environment. Participation is defined as the “ability to perform actions, tasks, and activities related to self-care, home management, work, community, and leisure roles in both socio-cultural and environmental contexts” (2). Participation restriction is associated with financial insecurity, malnutrition, increased healthcare cost, poor quality of life, frailty, institutionalization, and mortality (3–5). One of the biggest limitations of the ICF framework is the lack of a standardized tool to measure activity limitation and participation restriction (1, 6). Life-space mobility (LSM) is an important indicator of participation restriction (7–9). It is defined as the geographical area within which a person travels over a specified period in daily life, extent and frequency of movement, and any assistance needed (9). It depends on multiple complex interrelated factors such as physical function, ability to transfer, cognition, transportation, environment accessibility, vision, frailty, depression, nutrition, hearing impairment, falls, and fear of falling (FOF), which are all highly prevalent in an aging population (5, 7, 8, 10–13).

Falls and fear of falling (FOF) amongst older adults are common issues in countries with rapidly aging populations. Up to one-third of older adults ≥65 years fall each year in Canada and the United States, half of whom may experience recurrent falls, and falls accounts for nine in ten of all fractures in older adults (14, 15). FOF on the other hand affects between 20 and 85% of older adults, with significant threat to autonomy and participation (16, 17). Both falls and FOF are associated with social isolation, depression, anxiety, poor quality of life, and cognitive impairment, which result in participation restriction that imposes significant burden on the society and healthcare system at large (15, 18, 19). Risk factors common to both participation restriction, and falls and FOF are older age, low socio-economic status, gender where FOF is more prevalent in females, poor perceived health, frailty, and comorbidities (15, 20, 21).

Functional status is central to falls, healthy aging, and to the ability of individuals to move safely within their homes and across their environment. In 2015, the World Health Organization (WHO) defined “healthy aging” as “the process of developing and maintaining functional ability that enables wellbeing in older age” (22). The ability to function is determined by a complex relation between older adults' intrinsic capacity (IC) and their environment (23). IC is considered as a positive attribute and physiological reserve. Decline in IC with aging can lead to vicious cycle of sedentary behavior, loss of muscle mass, frailty, functional decline, FOF, and falls, all of which can cause participation restriction (24). The Integrated Care for Older People (ICOPE) care pathway by the WHO recommends screening for decline in IC through measurement of vision, hearing, cognition, nutrition, mobility, and mood, followed by person-centered assessment and development of personalized care and a monitoring plan to delay disability and dependency (22, 25). To enable active aging and participation, the WHO has also published a guide to Global Age-Friendly Cities for national stakeholders, emphasizing age-friendly living environments (transportation, housing, outdoor spaces, and buildings), social aspects (participation, respect, and inclusion), employment, community support, and health services (26).

Individual determinants, focusing on single factors such as activities of daily living (ADL), instrumental activities of daily living (IADL), gait speed, frailty, handgrip strength (HGS), falls, and LSM, have been used to assess mobility and/or participation and are associated with adverse outcomes. However, in real life, mobility and participation are dependent on a cluster of multiple independent interacting factors, most of which may be dynamic, such as physical function and frailty. Methodologies such as latent cluster analysis (LCA) profile can be useful to uncover subpopulations sharing similar characteristics or outcomes, and this can assist in designing future multi-domain prevention-intervention measures.

The aim of this study is threefold. First, we used the LCA to determine participation patterns amongst older adults with falls or at risk of falls. Second, we examined the association of participation patterns with the IC domains. Third, we developed a risk prediction model of IC impairments with the different participation groups.

## Method

### Study design and participants

This was a cross sectional study consisting of 155 community-dwelling older adults with falls or near-falls recruited for a falls prevention intervention study. Inclusion criteria were: (1) community-dwelling ≥60 years of age and (2) history of falls or near-falls in the past 12 months. Participants had to be able to understand, communicate, and provide informed consent. Participants were excluded if they had underlying severe cognitive impairment, were wheelchair bound, bedridden, or nursing-home residents. We restricted our analysis to 154 participants with complete information on all life-space variables. Written consent was obtained from all recruited participants, and protocol was approved by the National Healthcare Group (NHG), Domain Specific Review Board (DSRB), Singapore. The study complies with the Declaration of Helsinki ethical principles.

### Intrinsic capacity

The six IC domains were used to predict participation patterns amongst older adults at risk of falls. IC framework was first proposed by the WHO and released as the “Integrated care for older people: guidelines on community-level interventions to manage declines in intrinsic capacity” in 2017 (27), followed by the “Handbook: Guidance on person-centered assessment and pathways in primary care” in 2019 with a digital app for community-level screening and intervention (25). The recommendations include screening for cognition, vitality (nutrition), mobility [five times sit-to-stand (STS)], depression, and hearing and vision impairment (28). The Montreal Cognitive Assessment (MoCA) was used to assess cognitive status, and a cut off score of <26 was used to define cognitive impairment (29). Impaired STS was defined as >14 s. Performance of 6/18 or worse in one or both eyes was classified as vision impairment (30). For vitality, anorexia of aging was measured using the four-item Simplified Nutritional Appetite Questionnaire (SNAQ), where a score ≤14 indicated significant risk of at least 5% weight loss within 6 months (31). Hearing impairment was assessed by asking “Do you or your family think you may have hearing loss?”.

### Demographic and covariates

The interview questionnaire was administered by trained research assistants on demographics, chronic diseases, education, falls, physical function, physical activity, cognition, frailty, sarcopenia, depression, self-assessed health status, anorexia of aging, fear of falling, Falls Risk for Older People in the Community (FROP-Com), and LSM. The FRAIL (fatigue, resistance, aerobic, number of illnesses, and loss of weight) scale was used to assess frailty with a maximum score of 5. Pre-frail was defined as 1–2, frail 3–5, and robust 0 (32). Sarcopenia was screened using the SARC-F (lifting and carrying 10 pounds, walking across a room, transferring from bed/chair, climbing a flight of ten stairs, and frequency of falls in the past year), with scores ranging from 0 to 10, and ≥4 points was classified as having sarcopenia (33). ADL was assessed using the Katz ADL scale and IADL using Lawton's IADL scale (34, 35). Depression was assessed using the 15-item Geriatric Depression Scale (GDS), and a cut-off of ≥5 was used to define depression (36). Self-assessed health status was rated as excellent, very good, good, fair, or poor (37).

The Rapid Assessment of Physical Activity tool was used to assess physical activity (38). Fear of falling was assessed using the Falls Efficacy Scale-International (FES-I). This is based on self-reported concern of fear of falling while performing 16 ADLs such as getting dressed and going up or down the stairs). Each of the ADLs were scored based on “not concerned at all” to “very concerned”, with total scores ranging from 16 to 64 (39). The FROP-Com screening tool is a comprehensive falls risk assessment tool developed by the National Aging Research Institute Australia (40). It includes 13 risk factors with total scores ranging from 0 to 60, where 19–60 is considered high risk, 12-18 moderate risk, and 0–11 low risk (41). Social isolation was measured using the six-item Lubben Social Network Scale (LSNS-6). It measures size, closeness, and frequency of contact with friends and family members with total scale score ranging from 0 to 30 obtained by summing the six items, and a score below 12 was classified as at risk of social isolation (42). Loneliness was measured using the three-item UCLA scale with scores ranging from 3 to 9, where those scoring 3–5 were classified as “not lonely” and 6–9 as “lonely” (43).

Physical performance test included assessment of HGS, gait speed (4 meters), timed up-and-go (TUG), and short physical performance battery test (SPPB). HGS was measured using Jamar hand dynamometer on the dominant arm in the seated position with elbow flexed at 90°, and maximum HGS was recorded. Poor HGS was based on cut-offs of 28 kg for males and 18 kg for female as defined by the 2019 Asian Working Group for Sarcopenia (44). The SPPB included three components (balance, gait speed, and chair stand) with a maximum score of 12 points (4 points per-component).

### Life-space mobility

LSM was measured using the validated University of Alabama at Birmingham Life-Space Assessment questionnaire which was developed in 2003. LSM measures the ability of an individual to move within their own home and across the environment or region, and is quantified using the life-space mobility index (9, 10). The composite score is measured based on movement within home, neighborhood, or across the region, frequency (less than once a week, 1–3 times a week, 4–6 times a week, or daily), and use of assistance (walking aid and/or human) 4 weeks prior to the assessment. The scores range from 0 to 120, where 0 indicates activity may be limited to bedroom and 120 which indicates participant was fully mobile without aid outside his/her city.

### Patterns of participation

Patterns of participation of older adults are dependent on a bundle of variables which share similar outcome such as sedentary lifestyle, decline in physical function, institutionalization, and mortality. The participation patterns were modeled on related but independent factors such as ADL, IADL, gait speed, HGS, frailty, falls, FOF, education, and self-assessment of health status.

### Statistical analyses

Analyses were performed using STATA 17.0, with statistical significance set at *p* < 0.05. LCA was used to explore the number of participation clusters. The characteristics of individuals in the different participation clusters were compared using chi-square test for categorical variables and *T*-test for continuous variables. Logistic regression analysis was performed to assess the association between each of the IC domain (hearing, vision, cognition, mobility (poor STS) and nutrition (anorexia of aging)) and the participation clusters. Odds ratios with 95% confidence intervals (CI) were reported. Prediction models on participation typology were explored using ROC curve to evaluate their discriminative abilities.

## Results

### Development of participation patterns using LCA

Variables from previously published studies were used to explore the number of participation clusters, such as frailty, perceived health, falls, ADL, IADL, poor HGS, gait speed, LSM, FES-I, TUG, age, and years of education (4, 9, 13, 45–47). One to four participation clusters were examined and based on the lowest Consistent Akaike Information Criterion (AIC) and Bayesian-Schwarz Information Criterion (BIC) (9); a three-cluster solution was considered to be optimal (Table 1). Lower AIC and BIC values indicate a better model fit.

**Table 1:** Latent class analysis: Akaike's information criterion (AIC) and Bayesian information criterion (BIC).

**Number of clusters**	**AIC**	**BIC**
1	6,750	6,808
2	6,550	6,650
**3**	**6,430**	**6,567**
4	6,494	6,670

Bold implies statistical significance.

### Co-variates, LCA, and participation patterns

One hundred and fifty-four participants were divided into three participation clusters: high (*n* = 63, 40.9%), moderate (*n* = 83, 53.9%) and low (*n* = 8, 5.2%) (Table 2). Mean age was 74.62 ± 7.96 years, 35.7% were male, and average education was 5.57 ± 4.62 years. For functional status, 9.1% were frail and 50.0% pre-frail, 68.2% had poor HGS, 39.0% had ≥ 1 IADL, and 18.2% ≥ 1 ADL impairments. Mean LSM score was 63.60 ± 23.06, gait speed 0.83 ± 0.30 m/s, FES-I 21.73 ± 6.60, and TUG 13.75 ± 8.68 s. The high participation group compared with moderate and low participation groups were significantly younger: 71.19 ± 6.71, 77.00 ± 7.76, and 77.00 ± 10.12 years, respectively. Education level was highest in the low participation (7.75 ± 1.98 years) and high participation group (6.96 ± 4.68), followed by moderate participation group (4.27 ± 4.39). Half of the low participation group were frail, and the remaining half were pre-frail. On the other hand, there were no frail participants in the high participation group, where 41.3% were pre-frail and 58.7% robust. Functionally, three-quarters of the low participation group had at least one ADL and/or IADL limitation. Amongst the high participation group, only 17.5% had at least one ADL limitation and 4.8% at least one IADL limitation. There were no significant gender differences between the groups.

**Table 2:** Latent class analysis co-variates by participation group.

**Variables**	**All subjects** ** (*n* = 154)**	**Participation group**			***P*-value**
		**High** ** (*n* = 63; 40.9%)**	**Moderate** ** (*n* = 83; 53.9%)**	**Low** ** (*n* = 8;5.2%)**	
Age (years) (range)	74.62 ± 7.96 (60 to 100)	71.19 ± 6.71 (60 to 87)	77.00 ± 7.76 (60 to 100)	77.00 ± 10.12 (64 to 93)	< 0.001
Education years (range)	5.57 ± 4.62 (0 to 19)	6.96 ± 4.68 (0 to 19)	4.27 ± 4.39 (0 to 16)	7.75 ± 1.98 (6 to 10)	0.001
Frailty					< 0.001
Robust	63 (40.9)	37 858.7)	26 (31.3)	0 (0.0)	
Pre-frail	77 (50.0)	26 (41.3)	47 (56.6)	4 (50.0)	
Frail	14 (9.1)	0 (0.0)	10 (12.0)	4 (50.0)	
General Health					0.051
Good	110 (71.9)	51 (81.0)	55 (67.1)	4 (50.0)	
Fair/Poor	43 (28.1)	12 (19.0)	27 (32.9)	4 (50.0)	
Falls	99 (64.3)	38 (60.3)	58 (69.9)	3 (37.5)	0.126
≥1 IADL limitation	60 (39.0)	11 (17.5)	45 (51.8)	6 (75.0)	< 0.001
≥1 ADL limitation	28 (18.2)	3 (4.8)	19 (22.9)	6 (75.0)	< 0.001
Poor handgrip strength	105 (68.2)	28 (44.4)	70 (84.3)	7 (87.5)	< 0.001
Gait speed (m/s) (range)	0.83 ± 0.30 (0.14 to 1.72)	1.06 ± 0.22 (0.65 to 1.72)	0.69 ± 0.20 (0.15 to 1.23)	0.32 ± 0.13 (0.14 to 0.49)	< 0.001
Life-space mobility (range)	63.60 ± 23.06 (6 to 120)	81.41 ± 16.51 (46 to 120)	52.91 ± 17.74 (21 to 110)	34.19 ± 17.94 (6 to 69)	< 0.001
Falls-efficacy scale (range)	21.73 ± 6.60 (16 to 61)	19.65 ± 3.82 (16 to 31)	21.70 ± 5.11 (16 to 37)	38.50 ± 12.62 (24 to 61)	< 0.001
Timed up-and-go (range)	13.75 ± 8.68 (3.39 to 75.61)	9.18 ± 2.36 (8.31 to 30.72)	14.68 ± 4.03 (3.39 to 75.61)	43.93 ± 16.76 (26.58 to 75.61)	< 0.001

Values are *n* (%), mean ± sd. IADL, instrumental activity of daily living; ADL, activity of daily living.

Low HGS prevalence was highest in the low participation group (87.5%), followed by moderate participation (84.3%) and high participation groups (44.4%). High participation group had the highest LSM score (81.41 ± 16.51) compared with moderate participation (52.91 ± 17.74) and low participation groups (34.19 ± 17.94). FOF depicted by FES-I score was significantly higher in the low participation group compared with moderate or high participation groups: 38.50 ± 12.62, 21.70 ± 5.11, and 19.65 ± 3.82, respectively. Gait speed was significantly lower in the low participation group compared with moderate and high participation groups: 0.32 ± 0.13 m/s, 0.69 ± 0.20 m/s, and 1.06 ± 0.22, respectively. TUG was significantly prolonged in the low participation group. Other variables included in the LCA, but non-significant, included general health status and ≥ 1 falls.

### Background characteristics of study participants

Background characteristics of participants are shown in Table 3. Almost three-quarters (73.4%) of the participants were of Chinese ethnicity, and one-third (35.7%) was male. Amongst the participants, 10.4% were still working, 71.9% rated health status as good, and 64.3% had had one or more falls. For self-reported chronic diseases, 40.4% had diabetes, 70.8% hypertension, and 18.2% high cholesterol. The prevalence of sarcopenia based on SARC-F was 16.9%. Mean SPPB was 8.53 ± 2.73 and FROP-COM 10.56 ± 5.35.

**Table 3:** Demographic co-variates by participation group.

**Variables**	***n***	**All**	**Participation group**			***P*-value**
			**High**	**Moderate**	**Low**	
Males	154	55 (35.7)	23 (36.5)	29 (34.9)	3 (37.5)	0.975
Ethnicity	154					0.300
Chinese		113 (73.4)	53 (84.1)	55 (66.3)	5 (62.5)	
Malay		18 (11.7)	4 (6.3)	13 (15.7)	1 (12.5)	
Indian		20 (13.0)	5 (7.9)	13 (15.7)	2 (25.0)	
Others		3 (1.9)	1 (1.6)	2 (2.4)	0 (0.0)	
Still married	154	51 (33.1)	19 (30.3)	29 (34.9)	3 (37.5)	0.195
Still working	154	16 (10.4)	14 (22.2)	2 (2.4)	0 (0.0)	< 0.001
Lonely	154	30 (19.5)	6 (9.5)	21 (25.3)	3 (37.3)	0.024
Social isolation	154	79 (51.3)	26 (41.3)	50 (60.2)	3 (37.5)	0.050
Sarcopenia	154	26 (16.9)	1 (1.6)	21 (25.3)	4 (50.0)	< 0.001
Hypertension	128	92 (70.8)	33 (60.0)	55 (82.1)	4 (66.7)	0.025
High cholesterol	120	85 (18.2)	31 (64.4)	50 (75.8)	4 (66.7)	0.421
Diabetes	109	44 (40.4)	10 (22.2)	32 (55.2)	2 (33.3)	0.003
Stroke	90	13 (14.4)	5 (11.9)	6 (14.0)	2 (40.0)	0.238
BMI (kg/m^2^) (range)	151	24.58 ± 4.71 (13.77 to 46.83)	24.02 ± 4.41 (13.77 to 34.52)	25.11 ± 5.04 (15.40 to 46.83)	23.73 ± 3.23 (19.38 to 29.95)	0.344
SPBB (range)	154	8.53 ± 2.73 (0 to 12)	10.51 ± 1.49 (6 to 12)	7.52 ± 2.32 (0 to 12)	3.38 ± 1.30 (1 to 5)	< 0.001
FROP-COM (range)	154	10.56 ± 5.35 (1 to 26)	7.78 ± 4.61 (1 to 26)	12.01 ± 4.67 (4 to 24)	17.38 ± 5.85 (7 to 26)	0.001

Values are *n* (%), mean ± sd.

BMI, body mass index; SPPB, short physical performance battery test; FROP-COM, Falls Risk for Older People in the Community.

There were significant demographic differences between the three participation groups, where 22.2% of the high participation group were still working, 2.4% in the moderate participation and none in the low participation groups. Sarcopenia prevalence was 50% in the low participant group compared with 25.3% in the moderate and 1.6% in the high participation groups. For chronic diseases, hypertension prevalence was highest in the moderate participation group, followed by low participation and high participation groups: 82.1, 66.7, and 60%, respectively. Similarly, diabetes prevalence was highest in the moderate participation group (55.2%) and lowest in the high participation group (22.2%). SPPB scores were highest in the high participation group (10.51 ± 1.49) and lowest in the low participation group (3.38 ± 1.30). The low participation group had significantly higher FROP-Com scores compared with the high participation group (17.38 ± 5.85 vs. 7.78 ± 4.61, respectively).

### Relationship between participation groups and intrinsic capacity

Table 4 shows the prevalence of each IC component and its relationship with the latent participation classes. The prevalence of low cognition defined by MoCA<26 was 59.9%, anorexia of aging 29.9%, poor STS 57.2%, hearing loss 21.6%, visual impairment 78.5%, and depression 27.9%. In the unadjusted model with high participation as a reference category, moderate participation was associated with MoCA <26 (OR 8.1, 95% CI 3.8–17.3; *p* < 0.001), poor STS (OR 8.2, 95% CI 3.8–17.3; *p* < 0.001), hearing loss (OR 3.6, 95% CI 1.4–9.4; *p* = 0.010), and depression (OR 4.5, 95% CI 1.9–10.7; *p* = 0.001). In the adjusted model, only low MoCA scores (OR 4.2, 95% CI 1.7–10.4; *p* = 0.02) and poor STS (OR 7.1, 95% CI 3.0–17.0, *p* = < 0.001) remained significant. In the unadjusted model with high participation as a reference category, low participation was associated with anorexia of aging (OR 5.3, 95% CI 1.1–25.0; *p* < 0.034), poor STS (OR 17.5, 95% CI 2.0–187.5; *p* < 0.011), and hearing loss (OR 9.3, 95% CI 1.8–47.2; *p* = 0.07). In the adjusted model, low participation was associated with anorexia of aging (OR 9.9, 95% CI 1.6–60.9; *p* = 0.014), poor STS (OR 19.1, 95% CI 2.0–187.5; *p* = 0.011), and hearing loss (OR 9.8, 95% CI 1.4–70.8, *p* = 0.024).

**Table 4:** Intrinsic capacity and association with participation group.

**Variables**	**All subjects**	**Participation group**										
		**High**	**Moderate**					**Low**				
				**Unadjusted OR (95% CI)**	***P*-value**	**Adjusted OR (95% CI)**	***P*-value**		**Unadjusted OR (95% CI)**	***P*-value**	**Adjusted OR (95% CI)**	***P*-value**
MocA < 26	91/142 (59.9)	21/63 (33.3)	65/81 (80.2)	**8.1** **(3.8–17.3)**	**< 0.001**	**4.2** **(1.7–10.4)**	**0.002**	5/8 (62.5)	3.3 (0.73**–**15.3)	0.122	2.0 (0.32**–**12.9)	0.457
Anorexia	46/154 (29.9)	15/63 (23.8)	26/83 (31.3)	1.5 (0.70**–**3.1)	0.318	1.7 (0.65**–**4.6)	0.269	5/8 (62.5)	**5.3** **(1.1–25.0)**	**0.034**	**9.9** **(1.6–60.9)**	**0.014**
Poor STS	87/152 (57.2)	18/63 (28.6)	62/81 (76.5)	**8.2** **(3.8–17.3)**	**< 0.001**	**7.1** **(3.0–17.0)**	**< 0.001**	7/8 (87.5)	**17.5** **(2.0–152.6)**	**0.010**	**19.1** **(2.0–187.5)**	**0.011**
Hearing impairment	33/153 (21.6)	6/62 (9.7)	23/83 (27.7)	**3.6** **(1.4–9.4)**	**0.010**	2.1 (0.65**–**7.0)	0.215	4/8 (50.0)	**9.3** **(1.8–47.2)**	**0.007**	**9.8** **(1.4–70.8)**	**0.024**
Visual impairment	113/144 (78.5%)	43 /60 (71.7)	65/76 (57.5)	2.3 (0.99**–**5.5)	0.051	1.7 (0.60**–**5.1)	0.308	5/8 (62.5)	0.66 (0.14**–**3.1)	0.595	0.34 (0.05**–**2.2)	0.262
Depression	43/154 (27.9)	8/63 (12.7)	33/83 (39.8)	**4.5** **(1.9–10.7)**	**0.001**	1.8 (0.63**–**5.3)	0.266	2/8 (25.0)	2.3 (0.39**–**13.4)	0.357	1.0 (0.14**–**7.4)	0.998

Reference category: high participation.

Bold implies statistical significance.

### Prediction model for relationship between participation groups and intrinsic capacity

Three prediction models were investigated: model A, using the saved probabilities of the six IC domains from a logistic regression on low participation; model B, using the odds ratios from model A as the weighted scores and a simple model C, using 1 point for each positive IC (Table 5). There was no statistical significance across the three models. For simple clinical usage, Model C (named **MASHED**: ***M***ocA < 26, Anorexia of aging, Poor STS, Hearing Loss, Eye, Depression) was used (Table 6A). A participant with a decline in at least three out of the six IC domains had a probability of greater than 80% (72/88) to belong to the low/moderate participation group, with area under the curve (AUC) of 0.828 (95% CI 0.761–0.894, *p* < 0.001) (Table 6B).

**Table 5:** Prediction models.

**Model**	**AUC (95% CI)**	***P*** **-value**	
A	0.795 (0.719–0.870)	Compared to A	
B	0.792 (0.717–0.868)	0.484	Compared to B
C	0.781 (0.707–0.855)	0.138	0.165

Model A: Using the saved probabilities of the six intrinsic variables on low participation.

Model B: Using the odds ratios of Model A as weighted scores.

Model C: Adding up the number of positive intrinsic variables.

**Table 6A:** MASHED score (from intrinsic capacity) and participation group.

**MASHED Score (no. of intrinsic capacity domains)**	**All subjects** **(*n* = 154)**	**Participation group**			
		**High (*n* = 63)**	**Moderate (*n* = 83)**	**Low (*n* = 8)**	***P*-value**
0	6 (3.9)	6 (9.5)	0 (0.0)	0 (0.0)	
1	30 (19.5)	24 (38.1)	6 (7.2)	0 (0.0)	
2	30 (19.5)	17 (27.0)	12 (14.5)	1 (12.5)	
3	48 (31.2)	13 (20.6)	32 (38.6)	3 (37.5)	
4	22 (14.3)	1 (1.6)	18 (21.7)	3 (37.5)	
5	17 (11.0)	2 (3.2)	14 (16.9)	1 (12.5)	
6	1 (0.6)	0 (0.0)	1 (1.2)	0 (0.0)	
Mean ± SD	2.68 ± 1.37	1.76 ± 1.13	3.30 ± 1.17	3.50 ± 0.93	*p* < 0.001

**Table 6B:** Prediction model and participation group.

**Cut offs**	**Sensitivity (%)**	**Specificity (%)**	**PPV (%)**	**NPV (%)**
**Prediction for low participation (AUC** **=** **0.692, 95% CI 0.550–0.834**,				
***p*** **=** **0.068)**				
1	100	4.1	5.4	100
2	100	24.7	6.8	100
3 (Youden Index)	87.5	44.5	8.0	98.5
4	50.0	75.3	10.0	96.5
5	12.5	88.4	5.6	94.9
**Prediction for moderate/low participation (AUC** **=** **0.828, 95% CI 0.761–0.894**,				
***p*** **<** **0.001)**				
1	100	9.5	61.5	100
2	93.4	47.6	72.0	83.3
3 (Youden Index)	79.1	74.6	81.8	71.2
4	40.7	95.2	92.5	52.6
5	17.6	96.8	88.9	44.9

Values are *n* (%).

PPV, Positive Predictive Value; NPV, Negative Predictive value.

## Discussion

Using LCA, we were able to classify older adults at risk of falls as belonging to one of the three participation groups—high, moderate, and low. Participants in the high participation group were significantly younger, had less functional limitations, were more robust, and had better LSM and lower fear of falling. Compared to the high participation group, the low participation group was significantly associated with anorexia of aging, poor STS, and hearing loss, whereas the moderate participation group was significantly associated with poor STS and low cognition. Participants with at least three IC-domain impairments had a higher probability of belonging to the moderate or low participation groups.

Prior studies on participation have focused mainly on LSM with an arbitrary cut off of LSM <60 or ADL and/or IADL limitation to define low participation, which may be associated with a higher false negative rate compared with LCA used in our study (8). LSM is an important indicator for participation restriction and is shown to correlate with physical performance including activity of daily living (ADL), cognition, FOF, falls, quality of life, frailty, healthcare utilization, undernutrition, and mortality (4, 45–47). A recent study published by Watanabe et al. showed an L-shaped relationship between LSM and mortality over 5 years with dose–response relationship up to a score of 60 points (13). In our study, both the moderate and low participation groups had a mean LSM score of below 60. Falls lead to FOF, social isolation, depression, declining physical function, institutionalization, and mortality, all of which are risk factors for participation restrictions. Based on FROP-COM scores, both the low and moderate participation groups were categorized as at a moderate fall risk and would benefit from targeted personalized interventions (48).

LSM is vital for participation in social life and social inclusion, which was evident from our study, where individuals in the low participation group were three times more likely to be lonely compared with high participation group. Although not statistically significant, six in ten of the moderate participation group were at risk of social isolation compared with four in ten of the high participation group. LSM is also associated with poor health-related quality of life which was evident in our study participants, where only half of the low participation group rated their health as good compared with more than three-quarters of the high participation group (49). Frailty is a state of decreased physiological reserve, dynamic, multidimensional, and is associated with negative outcomes including falls, hospitalization, institutionalization, and mortality (50). Similar to other studies, frailty was significantly more prevalent in the low and moderate participation groups (51). Constricted LSM is a risk factor for frailty, and frailty predicts steeper decline in LSM. As frailty is reversible before the onset of disability, upstream screening and intervention is recommended before the onset of disability (46, 49, 51).

The prevalence of at least one IC impairment in our study population was 96.1%. In another study where the participants were mainly those with memory complaints, the prevalence of one IC impairment was 89.3% (52). The WHO ICOPE pathway recommends screening for IC, which has proven to be both feasible and practical and can be performed within a few minutes by trained community partners and/or healthcare professionals. It serves as a trigger for comprehensive assessment of underlying causes and intervention. ICOPE screening tools have shown to be able to identify older adults at high risk of progressing to frailty, ADL, and/or IADL disability (24, 53).

There is still ongoing debate on the ideal measurement model for intrinsic capacity, and there are no standardized assessment tools or intrinsic capacity composite score, e.g., IC index for clinical or research use (28). A recent scoping review highlighted that published studies use either a “formative” or “reflective” approach to conceptualize IC (54). The authors suggested that IC should be interpreted as a “system, which depends on the quantity and quality of the dynamical interrelations between its elements (capacities)”. The different domains could be interpreted as being separate but interacting entities that form an aggregated construct for physical and mental health, as decline in one domain can also affect impact on decline in another domain (54). González-Bautista et al. showed that impairment of each additional domain over 5 years increased risk of incident frailty by 47%, ADL impairment by 23%, and IADL impairment by 27% (53). Our study showed that participants with a decline in at least three out of the six intrinsic capacity domains had a probability of >80% of belonging to the low/moderate participation group. The benefits of prognostic scoring generated in our study can serve as a tool to stratify and prioritize risk interventions based on type and number of IC domains affected, which is relevant in clinical practice. Our study has further added to the literature that interventions to improve IC should target the different domains concurrently through multidomain interventions as suggested by the WHO (55).

Physical performance limitations defined by low gait speed, longer five times STS, and/or longer TUG was more prevalent in the low followed by moderate participation groups. Poor STS was significantly associated with moderate and low participation. The five times STS test is considered as a proxy-tool for physical performance and muscle strength in many international sarcopenia guidelines, although recent studies show that it is a better proxy of gait speed (physical performance) than HGS (muscle strength) (56).

Cognitive impairment was significantly associated with the low and moderate participation groups in our study population. Similar findings were shown in a systematic review, where prevalence of cognitive impairment was much higher in those with lower LSM (5). Cognition and participation share a complex and bidirectional relationship. It is known that participation may help preserve cognitive function and reduce the risk of dementia, and declining cognition is associated with low participation (5). Physical activity, gait speed, and social network are known to alter the trajectory of cognition and are also known risk factors for participation restriction.

Anorexia of aging is defined as decrease in food intake and/or appetite. It is a well-recognized precursor for malnutrition, loss of muscle mass, and frailty. Those who screened positive for anorexia of aging need to be evaluated for depression, loneliness, polypharmacy, access to food or inability to feed, swallowing disorder, or other chronic medical problems, all of which can be the cause or consequence of participation restriction (31). Earlier studies have shown significant association of LSM with nutrition (7, 10). Various recommendations to optimize dietary intake have been suggested in older adults at risk, including vitamin D supplementation, protein supplementation, and/or dietary modification to enhance nutrient density in combination with exercise, with positive impacts on frailty reversal, physical outcomes, and mobility (57).

Hearing impairment is known to affect many aspects of daily functioning and is a risk factor for disability, depression, activity limitation, and participation restriction mainly in those with severe/major hearing loss (58). Hearing impairment is prevalent in almost two-thirds of older adults and often under-reported in the old-old (59, 60). Compared with audiometric classification, 93.2% of participants ≥80 years old under-estimated hearing impairment when self-reporting (59). In our study individuals, prevalence of self-reported hearing loss was 21.6% and shown to be independently associated with low participation. Polku et al. similarly reported lower LSM scores in participants with major hearing difficulties, and those with mild or major hearing loss at baseline had significantly higher odds for restricted LSM at 2 years (61). While more studies are needed on the role of hearing aids in improving participation, the perceived benefit from hearing aids has been shown to be associated with better LSM scores (62). There was no significant difference in prevalence of vision impairment between the groups, possibly explained by the selection of participants with falls and/or near-falls where prevalence of vision impairment is known to be high. (14).

Prevalence of depression was highest in the moderate participation group, but its association was lost in the fully adjusted model suggesting that depression was secondary to other factors. Hill et al. showed that depressive symptoms associated with self-perceptions of memory problems contributed to lower physical and social participation (63). The low prevalence of depression in the low participation group and lack of association with participation group could be due to small sample size. Furthermore, depression is known to be highly prevalent in those with falls and near-falls (14).

Our study includes validated assessment tools for functional measures and targeted older adults with falls and near-falls. Unlike other studies which used LSM as an independent variable, our study used LCA comprising of factors associated with participation restriction. However, several limitations warrant mention. First and foremost, we acknowledge the small number of participants in the low participation group, which reflects the real-life scenario where they are the “extremes”. It is not known if intervention for anorexia of aging or hearing impairment may reduce the prevalence of low participation. For the exploratory analysis and prediction model, the low and moderate participation groups were combined. Other limitations include lack of objective hearing assessment using audiometry tests. Perceived health, demographics, history of falls, and life-space mobility measurement using questionnaire can be subject to recall bias (9). Objective vital parameters such as blood pressure together with self-reporting may be better indicators of perceived health. The study was conducted during the Covid-19 pandemic, which may have had an impact on overall life-space mobility. The cross-sectional study limits the causality association. In addition, our study population involved only those with falls or near-falls, and the findings from our study cannot be generalized to the population. We have no information on impact of environment on overall life-space mobility scores. However, most older adults in Singapore live in high-rise units in well-built neighborhoods. Lastly, there are no validated questionnaires to measure IC domains, and IC in this study was measured using a simple questionnaire which may be subject to recall bias.

Our study suggests that screening for impairments in IC in those with falls or at risk of falls will help identify older adults at risk of participation restriction especially those with poor STS, hearing impairment, cognitive impairment, and anorexia of aging. Although we have no data on interventions to improve participation and outcomes, most of the IC impairments may be reversible with targeted interventions. The recently published World Guidelines for Falls Prevention and Management for Older Adults have recommended multifactorial risk assessment, which includes all the IC domains with targeted interventions (64). Future longitudinal studies are needed at population level and to determine if interventions for IC decline will lead to improved participation and reduction in disability and frailty.

## Conclusion

Three distinct participation clusters were identified. The largest group was moderate participation, followed by high and low participation groups. Cognitive impairment and poor STS were significantly associated with moderate participation, while hearing impairment, anorexia of aging, and poor STS were associated with the low participation group. Screening for IC in those at risk of falls is important to develop a person-centered approach to promote increased participation. Future studies are needed at population level to assess the association of IC with participation and the impact of a personalized management plan on overall participation.

## Data availability statement

The raw data supporting the conclusions of this article will be made available by the authors, without undue reservation.

## Ethics statement

The studies involving human participants were reviewed and approved by National Healthcare Group Domain Specific Review Board, Singapore. The patients/participants provided their written informed consent to participate in this study.

## Author contributions

RM and YC contributed to study concept, design, and preparation of manuscript. RM conducted the data acquisition. YC conducted the data analysis and interpretation. RM, YC, IA, and JM were involved in writing and reviewing the manuscript. All authors contributed to the article and approved the submitted version.

## Funding

This study was funded by the Ministry of Health Singapore National Innovative Challenge on Active and Confident Aging Grant, MOH/NIC/F2/2017.

## Conflict of interest

The authors declare that the research was conducted in the absence of any commercial or financial relationships that could be construed as a potential conflict of interest.

## Publisher's note

All claims expressed in this article are solely those of the authors and do not necessarily represent those of their affiliated organizations, or those of the publisher, the editors and the reviewers. Any product that may be evaluated in this article, or claim that may be made by its manufacturer, is not guaranteed or endorsed by the publisher.

## References

[1] Castaneda L Bergmann A Bahia L. The international classification of functioning, disability and health: a systematic review of observational studies. Rev Bras Epidemiol. (2014) 17:437–51. 10.1590/1809-4503201400020012ENG 24918415

[2] International Classification of Functioning Disability Health. World Health Organization (2001). Available online at: https://apps.who.int/iris/handle/10665/42417 (accessed August 20, 2022).

[3] Lucas JW Schiller JS Benson V. *Summary health statistics for US adults: National Health Interview Survey, 2001*. National Center for Health Statistics. Vital Health Stat. (2004) 2004:1–134. 15791758

[4] Kennedy RE Williams CP Sawyer P Lo AX Connelly K Nassel A. Life-space predicts health care utilization in community-dwelling older adults. J Aging Health. (2017) 31:280–92. 10.1177/0898264317730487 29254407

[5] De Silva NA Gregory MA Venkateshan SS Verschoor CP Kuspinar A. Examining the association between life-space mobility and cognitive function in older adults: a systematic review. J Aging Res. (2019) 2019:3923574. 10.1155/2019/3923574 31275650 PMC6589294

[6] Darzins SW Imms C Di Stefano M. Measurement of activity limitations and participation restrictions: examination of ICF-linked content and scale properties of the FIM and PC-PART instruments. Disabil Rehabil. (2017) 39:1025–38. 10.3109/09638288.2016.1172670 27206817

[7] Johnson J Rodriguez MA Al Snih S. Life-space mobility in the elderly: current perspectives. Clinical Intervent Aging. (2020) 15:1665–74. 10.2147/CIA.S196944 32982200 PMC7501960

[8] Lee JQ Ding YY Latib A Tay L Ng YS. Intrinsic capacity and its relationship with life-space mobility (INCREASE): a cross-sectional study of community-dwelling older adults in Singapore. BMJ Open. (2021) 11: e054705. 10.1136/bmjopen-2021-054705

[9] Baker PS Bodner EV Allman RM. Measuring life-space mobility in community-dwelling older adults. J Am Geriatr Soc. (2003) 51:1610–4. 10.1046/j.1532-5415.2003.51512.x 14687391

[10] Kuspinar A Verschoor CP Beauchamp MK Dushoff J Ma J Amster E. Modifiable factors related to life-space mobility in community-dwelling older adults: results from the Canadian longitudinal study on aging. BMC Geriatrics. (2020) 20:1–2. 10.1186/s12877-020-1431-5 32005107 PMC6995110

[11] Fristedt S Kammerlind A-S Fransson EI Bravell ME. Physical functioning associated with life-space mobility in later life among men and women. BMC Geriatrics. (2022) 22:1–7. 10.1186/s12877-022-03065-9 35473475 PMC9040227

[12] Auais M Alvarado B Guerra R Curcio C Freeman EE Ylli A. Fear of falling and its association with life-space mobility of older adults: a cross-sectional analysis using data from five international sites. Age Ageing. (2017) 46:459–65. 10.1093/ageing/afw239 28043980 PMC5405754

[13] Watanabe D Yoshida T Yamada Y Watanabe Y Yamada M Fujita H. Dose-response relationship between life-space mobility and mortality in older Japanese adults: a prospective cohort study. J Am Med Dir Assoc. (2022). 23:1869.e7–1869.e18. 10.1016/j.jamda.2022.04.017 35636462

[14] Ambrose AF Cruz L Paul G. Falls and fractures: a systematic approach to screening and prevention. Maturitas. (2015) 82:85–93. 10.1016/j.maturitas.2015.06.035 26255681

[15] Thomas SM Parker A Fortune J Mitchell G Hezam A Jiang Y. Global evidence on falls and subsequent social isolation in older adults: a scoping review. BMJ Open. (2022) 12:e062124. 10.1136/bmjopen-2022-062124 36175106 PMC9528590

[16] Curcio CL Gomez F Reyes-Ortiz CA. Activity restriction related to fear of falling among older people in the Colombian Andes mountains: are functional or psychosocial risk factors more important? J Aging Health. (2009) 21:460–79. 10.1177/0898264308329024 19318606

[17] Scheffer AC Schuurmans MJ van Dijk N van der Hooft T de Rooij SE. Fear of falling: measurement strategy, prevalence, risk factors and consequences among older persons. Age Ageing. (2008) 37:19–24. 10.1093/ageing/afm169 18194967

[18] Wilkie R Peat G Thomas E Croft P. The prevalence of person-perceived participation restriction in community-dwelling older adults. Qual Life Res. (2006) 15:1471–9. 10.1007/s11136-006-0017-9 17009086

[19] Sheets KM Kats AM Langsetmo L Mackey D Fink HA Diem SJ. Life-space mobility and healthcare costs and utilization in older men. J Am Geriatr Soc. (2021) 69:2262–72. 10.1111/jgs.17187 33961699 PMC8542432

[20] Merchant RA Chen MZ Wong BLL Ng SE Shirooka H Lim JY. Relationship between fear of falling, fear-related activity restriction, frailty, and sarcopenia. J Am Geriatr Soc. (2020) 68:2602–8. 10.1111/jgs.16719 32804411

[21] Choi K Ko Y. Characteristics associated with fear of falling and activity restriction in South Korean older adults. J Aging Health. (2015) 27:1066–83. 10.1177/0898264315573519 25804899

[22] Beard JR Officer A de Carvalho IA Sadana R Pot AM Michel J-P. The World report on ageing and health: a policy framework for healthy ageing. Lancet. (2016) 387:2145–54. 10.1016/S0140-6736(15)00516-4 26520231 PMC4848186

[23] Beard JR Jotheeswaran AT Cesari M de Carvalho IA. The structure and predictive value of intrinsic capacity in a longitudinal study of ageing. BMJ Open. (2019) 9:e026119. 10.1136/bmjopen-2018-026119 31678933 PMC6830681

[24] Beard JR Si Y Liu Z Chenoweth L Hanewald K. Intrinsic capacity: validation of a new who concept for healthy aging in a longitudinal Chinese study. J Gerontol A Biol Sci Med Sci. (2022) 77:94–100. 10.1093/gerona/glab226 34343305

[25] World Health Organization. Integrated Care for Older People (ICOPE): Guidance for Person-Centred Assessment and Pathways in Primary Care. (2019). Available online at: https://www.who.int/publications/i/item/WHO-FWC-ALC-19.1 (accessed November 11, 2022).

[26] Plouffe L Kalache A. Towards global age-friendly cities: determining urban features that promote active aging. J Urban Health. (2010) 87:733–9. 10.1007/s11524-010-9466-0 20549569 PMC2937125

[27] World Health Organization. Integrated Care for Older People: Guidelines on Community-level Interventions to Manage Declines in Intrinsic Capacity (2017). Available online at: https://www.who.int/publications/i/item/9789241550109 (accessed August 20, 2022). 29608259

[28] George PP Lun P Ong SP Lim WS. A rapid review of the measurement of intrinsic capacity in older adults. J Nutr Health Aging. (2021) 25:774–82. 10.1007/s12603-021-1622-6 34179933 PMC7966899

[29] Dong Y Lee WY Basri NA Collinson SL Merchant RA Venketasubramanian N. The montreal cognitive assessment is superior to the mini–mental state examination in detecting patients at higher risk of dementia. Int Psychogeriatrics. (2012) 24:1749–55. 10.1017/S1041610212001068 22687278

[30] WHO. Blindness and vision impairment. (2021). Available online at: https://www.who.int/news-room/fact-sheets/detail/blindness-and-visual-impairment (accessed August 20, 2022).

[31] MerchantRAWooJMorleyJE. Editorial: anorexia of ageing: pathway to frailty and sarcopenia. J Nutr Health Aging. (2022) 26:3–5. 10.1007/s12603-021-1703-635067696

[32] Morley JE Malmstrom TK Miller DK. A simple frailty questionnaire (FRAIL) predicts outcomes in middle aged African Americans. J Nutr Health Aging. (2012) 16:601–8. 10.1007/s12603-012-0084-2 22836700 PMC4515112

[33] Malmstrom TK Morley JE. SARC-F: a simple questionnaire to rapidly diagnose sarcopenia. J Am Med Dir Assoc. (2013) 14:531–2. 10.1016/j.jamda.2013.05.018 23810110

[34] Wallace M Shelkey M. Katz index of independence in activities of daily living (ADL). Urol Nurs. (2007) 27:93–4. 17390935

[35] Lawton M. Assessment of older people: self-maintaining and instrumental activities of daily living. Gerontologist. (1969) 9:179–86. 10.1093/geront/9.3_Part_1.179 5349366

[36] de Craen AJ Heeren T Gussekloo J. Accuracy of the 15-item geriatric depression scale (GDS-15) in a community sample of the oldest old. Int J Geriatr Psychiatry. (2003) 18:63–6. 10.1002/gps.773 12497557

[37] Idler EL Kasl S. Health perceptions and survival: do global evaluations of health status really predict mortality? J Gerontol. (1991) 46:S55–65. 10.1093/geronj/46.2.S55 1997583

[38] Topolski TD LoGerfo J Patrick DL Williams B Walwick J Patrick MB. The rapid assessment of physical activity (RAPA) among older adults. Prev Chronic Dis. (2006) 3:A118. 16978493 PMC1779282

[39] Delbaere K Close JC Mikolaizak AS Sachdev PS Brodaty H Lord SR. The falls efficacy scale international (FES-I). A comprehensive longitudinal validation study. Age Ageing. (2010) 39:210–6. 10.1093/ageing/afp225 20061508

[40] Russell MA Hill KD Blackberry I Day LM Dharmage SC. The reliability and predictive accuracy of the falls risk for older people in the community assessment (FROP-Com) tool. Age Ageing. (2008) 37:634–9. 10.1093/ageing/afn129 18565980

[41] Falls Risk For Older People In The Community: FROP-COM. Available online at: https://www.nari.net.au/frop-com (accessed August 20, 2022).

[42] Lubben J Blozik E Gillmann G Iliffe S. Kruse Wv Beck JC. Performance of an abbreviated version of the lubben social network scale among three European community-dwelling older adult populations. Gerontologist. (2006) 46:503–13. 10.1093/geront/46.4.503 16921004

[43] Liu T Lu S Leung DKY Sze LCY Kwok WW Tang JYM. Adapting the UCLA 3-item loneliness scale for community-based depressive symptoms screening interview among older Chinese: a cross-sectional study. BMJ Open. (2020) 10:e041921. 10.1136/bmjopen-2020-041921 33303463 PMC7733209

[44] Chen L-K Woo J Assantachai P Auyeung T-W Chou M-Y Iijima K. Asian working group for sarcopenia: 2019 consensus update on sarcopenia diagnosis and treatment. J Am Med Dir Assoc. (2020). 10.1016/j.jamda.2019.12.012 32033882

[45] Portegijs E Rantakokko M Viljanen A Sipilä S Rantanen T. Identification of older people at risk of ADL disability using the life-space assessment: a longitudinal cohort study. J Am Med Dir Assoc. (2016) 17:410–4. 10.1016/j.jamda.2015.12.010 26805752

[46] Xue Q-L Fried LP Glass TA Laffan A Chaves PHM. Life-space constriction, development of frailty, and the competing risk of mortality: the women's health and aging study I. Am J Epidemiol. (2008) 167:240–8. 10.1093/aje/kwm270 17906296

[47] Lo AX Brown CJ Sawyer P Kennedy RE Allman RM. Life-space mobility declines associated with incident falls and fractures. J Am Geriatr Soc. (2014) 62:919–23. 10.1111/jgs.12787 24731095 PMC4049071

[48] Lee SM Loo G Long W Lock JZ Soh SY Seetharaman SK. Risk assessment and falls prevention in the older adult: Asian experience with the falls risk for older people in the community tool. Geriatr Gerontol Int. (2017) 17:518–9. 10.1111/ggi.12889 28345235

[49] Bentley JP Brown CJ Jr GM Sawyer P Allman RM Roth DL. Functional status, life-space mobility, and quality of life: a longitudinal mediation analysis. Quality Life Res. (2013) 22:1621–32. 10.1007/s11136-012-0315-3 23161329 PMC3618999

[50] Dent E Morley JE Cruz-Jentoft AJ Woodhouse L Rodríguez-Mañas L Fried LP. Physical frailty: ICFSR international clinical practice guidelines for identification and management. J Nutr Health Aging. (2019) 23:771–87. 10.1007/s12603-019-1273-z 31641726 PMC6800406

[51] Portegijs E Rantakokko M Viljanen A Sipilä S Rantanen T. Is frailty associated with life-space mobility and perceived autonomy in participation outdoors? A longitudinal study *Age Ageing*. (2016) 45:550–3. 10.1093/ageing/afw072 27126330

[52] González-Bautista E Barreto PD Giudici KV Andrieu S Rolland Y Vellas B. Frequency of conditions associated with declines in intrinsic capacity according to a screening tool in the context of integrated care for older people. J Frailty Aging. (2021) 10:94–102. 10.14283/jfa.2020.42 33575697

[53] González-Bautista E. Barreto PD, Andrieu S, Rolland Y, Vellas B. Screening for intrinsic capacity impairments as markers of increased risk of frailty and disability in the context of integrated care for older people: Secondary analysis of MAPT. Maturitas. (2021) 150:1–6. 10.1016/j.maturitas.2021.05.011 34274071

[54] Koivunen K Schaap LA Hoogendijk EO Schoonmade LJ Huisman M van Schoor NM. Exploring the conceptual framework and measurement model of intrinsic capacity defined by the World Health Organization: a scoping review. Ageing Res Rev. (2022) 10:101685. 10.1016/j.arr.2022.101685 35830956

[55] George PP Lun P Ong SP Lim WS. Operationalising the concept of intrinsic capacity in clinical settings. Background Paper for the WHO Working Group on Metrics and Research Standards for Healthy Ageing. (2017). p. 2–6.

[56] Ryu JY Kim M Kim KS Kim S Won CW. Chair stand test as a proxy for physical performance and muscle strength in sarcopenia diagnosis: the Korean frailty and aging cohort study. Aging Clin Exp Res. (2022) 34:2449–56. 10.1007/s40520-022-02172-2 35918606

[57] Lorbergs AL Prorok JC Holroyd-Leduc J Bouchard DR Giguere A Gramlich L. Nutrition and physical activity clinical practice guidelines for older adults living with frailty. J Frailty Aging. (2022) 11:3–11. 10.14283/jfa.2021.51 35122084

[58] Lin TC Yen M Liao YC. Hearing loss is a risk factor of disability in older adults: a systematic review. Arch Gerontol Geriatr. (2019) 85:103907. 10.1016/j.archger.2019.103907 31352184

[59] Kamil RJ Genther DJ Lin FR. Factors associated with the accuracy of subjective assessments of hearing impairment. Ear Hear. (2015) 36:164–7. 10.1097/AUD.0000000000000075 25158982 PMC4272625

[60] Lin FR Thorpe R Gordon-Salant S Ferrucci L. Hearing loss prevalence and risk factors among older adults in the United States. J Gerontol A Biol Sci Med Sci. (2011) 66:582–90. 10.1093/gerona/glr002 21357188 PMC3074958

[61] Polku H Mikkola TM Rantakokko M Portegijs E Törmäkangas T Rantanen T. | Self-reported hearing difficulties and changes in life-space mobility among community-dwelling older adults: a Two-year follow-Up study. BMC Geriatr. (2015) 15:121. 10.1186/s12877-015-0119-8 26459630 PMC4603343

[62] Polku H Mikkola TM Gagné J-P Rantakokko M Portegijs E Rantanen T. Perceived benefit from hearing aid use and life-space mobility among community-dwelling older adults. J Aging Health. (2018) 30:408–20. 10.1177/0898264316680435 27913763

[63] Hill NL Mogle J Bhargava S Bratlee-Whitaker E Wion RK Sweeder L. Within-person associations among self-perceptions of memory, depressive symptoms, and activity participation in older adults. Gerontologist. (2021) 61:1107–17. 10.1093/geront/gnaa208 33326557 PMC8437508

[64] Montero-Odasso M van der Velde N Martin FC Petrovic M Tan MP Ryg J. World guidelines for falls prevention and management for older adults: a global initiative. Age Ageing. (2022) 51: afac205. 36178003 10.1093/ageing/afac205 PMC9523684

